# FEF Excitability in Attentional Bias: A TMS-EEG Study

**DOI:** 10.3389/fnbeh.2018.00333

**Published:** 2019-01-11

**Authors:** Sara Torriero, Giulia Mattavelli, Emanuele Lo Gerfo, Leonor Romero Lauro, Rossana Actis-Grosso, Paola Ricciardelli

**Affiliations:** ^1^Department of Psychology, University of Milano—Bicocca, Milan, Italy; ^2^NeuroMi, Milan Center for Neuroscience, Milan, Italy; ^3^Department of Economics Management and Statistics, University of Milano—Bicocca, Milan, Italy

**Keywords:** frontal eye field, attention, face, competition, social, TMS, EEG

## Abstract

The role of distinct cortical regions in guiding social orienting needs further investigation. Our aim was to explore the contribution of the frontal eye field (FEF) in early orienting of attention towards stimuli with social value. We used a TMS-EEG approach to investigate event related potentials (ERPs; no-TMS block) and TMS evoked potentials (TEPs; TMS block) during the cueing phase of a modified version of the dot-probe task, comparing competing (face vs. house) and not competing (house vs. house) conditions. Our results revealed an increased amplitude of ERP components in the competing condition, showing greater posterior N170 and fronto-central vertex positive potential (VPP) and an enhanced frontal negative component at 250–270 ms from cue onset. TMS pulses over the FEF induced similar N170 and VPP amplified components. In addition, in the ERPs, a reduced positivity at 400 ms was shown when the face appeared on the left side vs. the right side of space. In contrast, in the TMS blocks, we found lateralized effects on N170 depending on the side of face presentation. The enhanced cortical excitability induced by TMS over the right FEF significantly correlated with the performance on the behavioral task, suggesting a link between the FEF activity during the cueing phase of the dot-probe task and the subsequent behavioral response times to the targets.

## Introduction

Humans are social beings whose adaptive behavior is crucially mediated both by attention and by the processing and understanding of social stimuli such as faces, body cues and gaze direction. Orienting attention allows the selection of the most behaviorally relevant stimuli by prioritizing their processing, a phenomenon that can be described as an attentional bias. This was first described as an automatic, stimulus-driven process triggered by the biological and social relevance of the eye gaze. However, current evidence shows that, in addition to gaze direction, attentional bias can be triggered by different types of stimuli (e.g., arrows, Tipples, 2002; Actis-Grosso and Ricciardelli, 2017) and modulated by several top-down and bottom-up factors (Liuzza et al., 2011; Ricciardelli et al., 2013; Ciardo et al., 2014).

Attentional bias has been extensively tested using a visuospatial “dot-probe task,” which showed faster orienting towards threat-related information among anxious populations (Mogg et al., 1994; Bar-Haim et al., 2007). Usually, in this task, a pair of facial expressions (cues) with different affective valences (e.g., angry/neutral, thus competing, or neutral/neutral, thus not competing) is presented to participants lateralized in the left or right visual field. Then, participants are asked to respond to a target that appears in the location previously occupied by one of the facial cues. Typically, faster orienting of attention is shown towards targets replacing emotional stimuli (congruent trials) than neutral stimuli (incongruent trials).

It has been proposed that limbic structures and top-down influences from the frontoparietal network to the visual cortex could play a role in controlling the allocation of attention towards social stimuli such as threat-related faces, while the ventromedial prefrontal region and the anterior cingulate cortex would be involved in controlling attention in conflict situations such as invalidly cued probes (Bush et al., 2000; Pourtois et al., 2006; Santesso et al., 2008). This hypothesis is supported by the influential neuroanatomical model of attention described by Corbetta and Shulman (2002), which defines two cortico-cortical neural systems involved in attending to environmental stimuli: a dorsal frontoparietal network, whose core regions include the dorso-parietal cortex and the dorso-frontal cortex broadly corresponding to the frontal eye field (FEF), engaged in top-down control mechanisms and a ventral frontoparietal network, including the temporoparietal junction (TPJ) and the ventral frontal cortex (VFC), which interacts with the dorsal frontoparietal network when attention is reoriented to behaviorally relevant stimuli (Corbetta et al., 2008; Chica et al., 2011; Vossel et al., 2014).

The initial involuntary orienting towards socially relevant stimuli during the cueing phase of the dot-probe task has been partially explored. The electrophysiological correlates of the orienting mechanism have been described as an enhancement of the early visual P1 activity in the extrastriate cortex in response to targets replacing fearful faces, preceded by modulatory activity in posterior parietal regions (Pourtois et al., 2004, 2005; Santesso et al., 2008). Event related potentials (ERPs) time-locked to face onset have been described, with different timing and scalp distributions (Torrence and Troup, 2018): the early C1 component, which has an onset latency of 50 ms following stimulus presentation and is thought to reflect initial activity of the primary visual cortex; the N170, a negative ERP component originating over occipito-temporal areas, peaking approximately at 170 ms after stimulus onset, which is consistently elicited by human faces (Bentin et al., 1996; Eimer, 2000; Rossion et al., 2000); and the N2pc, a negative posterior component occurring approximately 150–250 ms after cue onset contralateral to the stimulus presentation, thought to indicate initial orienting of attention (Luck and Hillyard, 1994; Diao et al., 2017). However, EEG studies that examined the neural responses evoked by emotional faces have reported contrasting results about the implication of these ERP components in response to threat-related faces and their modulation by attentional processes (Pourtois et al., 2004; Santesso et al., 2008; Holmes et al., 2009, 2014; Carlson and Reinke, 2010; Brosch et al., 2011).

Further EEG components described in attentional orienting are early direct attention negativity (EDAN) and anterior DAN (ADAN), which are known to mark the initial orienting and maintaining of attention, respectively (Nobre et al., 2000; Eimer et al., 2002; Brignani et al., 2009; Seiss et al., 2009; Praamstra and Kourtis, 2010). EDAN typically consists of a posterior negativity approximately 200–400 ms after cue onset over the hemisphere contralateral to the direction indicated by the cue, followed at later time intervals by a negativity over anterior scalp sites (ADAN; Talsma et al., 2005). These cue-locked ERP components are elicited for voluntary shifts of attention but also for noninformative task-irrelevant symbolic cues (Ranzini et al., 2009) and during reflexive attention shifting triggered by social cues, such as gaze (Feng and Zhang, 2014). Lassalle and Itier (2013) found that in a gaze-cueing paradigm, EDAN and ADAN components were not modulated by emotional valence. In contrast, an ERP study investigating attentional capture by red-colored images in an emotional context using a modified version of the dot-probe task (Kuniecki et al., 2015) showed a modulatory effect of the affective valence of the cues over the EDAN and ADAN components. The authors suggested that the EDAN component was influenced by both physical features of stimuli and by the rapid decoding of the images’ semantic meaning.

Social stimuli processing and attentional orienting recruit a widely distributed network implicated in face perception and in emotion and social cognition, including temporal, frontoparietal and subcortical networks (Haxby et al., 2000; Nummenmaa and Calder, 2009). Convergent research from animal models and studies on human clinical and healthy populations has highlighted the critical involvement of the prefrontal cortex in facial expression processing, gaze cueing and social cognition (Frischen et al., 2007; Browning et al., 2010b; Mattavelli et al., 2011, 2013, 2016; Wiese et al., 2018). Although previous studies have shown that top-down signals from the prefrontal cortex allow the control of overt and covert attentional processing, evidencing the role of the FEF in visual awareness, target discrimination and spatial orienting of attention (Grosbras and Paus, 2003; O’Shea et al., 2004; Ruff et al., 2006; Morishima et al., 2009; Marshall et al., 2015), little is known about the contribution of this frontal region in prioritizing attentional processes towards socially relevant stimuli, such as emotional faces.

In the present study, we aimed to clarify the contribution of the FEF in early orienting of spatial attention driven by stimuli with high social value. According to the hypothesis that high priority stimuli, such as social and emotional stimuli, bias attentional processing (Mather and Sutherland, 2011), the frontoparietal attention network modulates the activity of the posterior regions for faster perceptual processing and target detection. For this purpose, we created a modified version of the dot-probe task using stimuli with different social impacts as cues, namely, faces with angry expression vs. houses. Indeed, compared to other visual stimuli, faces automatically capture attention (Bindemann et al., 2007), and angry emotional expression strengthens their social meaning. To investigate the contribution of the FEF, we used an integrated TMS-EEG system, which allowed us to explore the timing and cortical distribution of brain excitability (Miniussi and Thut, 2010) induced by right FEF stimulation during the cueing phase of our task. TMS-EEG indeed allows direct perturbation of the cortical activity of any brain area, by means of TMS and recording with the EEG the cortical response to this perturbation with a high temporal resolution (Taylor et al., 2008) all over the cortex, thus unveiling how activation spreads from the stimulated area to the interconnected ones. Such cortical responses are called TMS evoked potentials (TEPs), and they are considered a reliable measure of the brain activation state and cortical effective connectivity (Taylor and Thut, 2012). Therefore, in addition to examining the role of the FEF, this approach allowed us to explore whether the cortical responsiveness in the attentional and face-related networks can be modulated by the competition between stimuli with different social connotations.

Since the FEF is known to be involved in the early stages of top-down control and attentional orienting (Corbetta et al., 2008; Vernet et al., 2014), we hypothesized that the attentional bias driven by competing cues would affect the cortical excitability induced by TMS. We predicted different cortical responsiveness for competing (angry face vs. house) and not-competing (house vs. house) pairs of cueing stimuli related to the attentional and face networks, which should be reflected in the attentional bias measured in the dot-probe task. In particular, we expected that the FEF perturbation by TMS would induce differences in cortical excitability in the early components related to the top-down activity of the frontoparietal attentional network.

We also hypothesized that the FEF excitability would be dependent on the side of presentation of the face stimuli due to the functional overlap of the neural mechanisms that control spatial attention and saccadic eye movements (Awh et al., 2006). We expected cortical responses associated with the orienting mechanisms of attention towards lateral stimuli and early responses to the right FEF perturbation related to the laterality of face presentation.

However, given the lack of homogeneity in previous studies regarding the EEG dynamics time-locked to faces in the dot-probe task and the specific role played by the FEF, we aimed to explore the general distribution of the neurophysiological response related to the cueing phase of the task. For this reason, we tested whole-head EEG responses over a large time window, avoiding* a priori* selection of expected EEG components.

## Materials and Methods

### Participants

Fifteen healthy participants (mean age = 25, range 22–32 years; three men) participated in the study. All participants were right-handed according to the Edinburgh Handedness Inventory, and none had contraindications to receive TMS (Rossi et al., 2009). This study was carried out in accordance with the recommendations of the American Psychological Association (APA). All participants gave written informed consent in accordance with the Declaration of Helsinki. The protocol was approved by the Ethics Committee of University of Milano—Bicocca.

### Materials

A modified version of the dot-probe task was used, with angry faces (F) and houses (H) as stimuli. Each experimental block consisted of 96 trials with angry face and house pairs (FH), which represented the competition condition, and 96 trials with only house pairs (HH), which served as the control condition (no competition). Eight different pictures of angry face expressions were selected from The Karolinska Directed Emotional Faces (KDEF) database (Lundqvist et al., 1998), and 24 different pictures of houses were selected from a free database^1^. Each face and house picture was repeated 12 times in each experimental block. Although in total faces were presented less frequently than houses, the effect of novelty was reduced by the inclusion of more exemplars of houses, so that each face and house picture was presented the same number of times. This is consistent with the stimulus selection adopted by Bradley et al. (1997) in their original pictorial version of the dot-probe task, in which they presented neutral stimuli more frequently than emotional stimuli, reducing the effect of novelty/habituation with the inclusion of different versions of the same neutral category (Staugaard, 2009).

**Figure 1:**
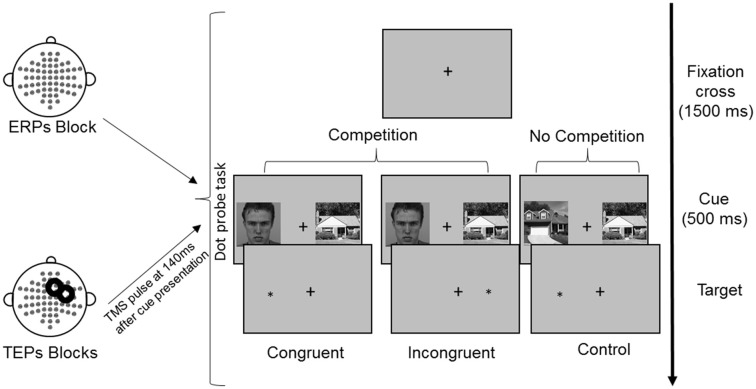
Experimental procedure and conditions of the dot-probe task. Following presentation of a fixation cross for 1,500 ms, cues were presented simultaneously for 500 ms. Reaction times (RTs) were measured from the onset of the target, which followed the cues until the button press. In TMS blocks, TMS was delivered over the right frontal eye field (FEF) at 140 ms from cue onset. The represented face picture is from the Karolinska Directed Emotional Faces database (AM08ANS).

All images were converted on a grayscale and resized at 325 × 325 pixels. The mean luminance was calculated for each image and adjusted to make it equal across images using IrfanView imaging software (Irfan Skiljan, Wiener Neustadt, Austria). Participants were seated in front of a computer screen at a distance of 120 cm. The selected images were simultaneously presented on a black background in the left and right visual field with a visual angle of 4°, separated by a central white cross. The image size measured 11.5 × 11.5 cm on the screen (4.4°). The side of faces and houses presentation (left or right) was balanced, as well as the congruency with the subsequent target (Figure 1). Each FH and HH pair, for each side of presentation and congruency condition, underwent three repetitions in each experimental block.

The task was presented on a 22″ pc monitor using E-prime software 2.0 (Psychology Software Tools, Pittsburgh, PA, USA), and responses were collected using a pc keyboard.

### Design and Procedure

The experimental paradigm consisted of a modified version of the dot-probe task (MacLeod et al., 1986), which is widely used to measure attention allocation to stimuli. The task is based on the finding that individuals tend to respond faster to a probe stimulus (e.g., a small dot) that is presented in an attended rather than unattended spatial area of the visual display (Posner et al., 1980; Navon and Margalit, 1983).

In each trial, a pair of cue stimuli (FH in the competition condition or HH in the no competition condition) was presented for 500 ms, followed by a probe, namely, a white asterisk that appeared in the location previously occupied by one of the cues. For the competition condition, in the congruent trials the probe appeared at the same location of faces, whereas in the incongruent trials the probe appeared at the opposite location (Figure 1). The probe remained on the screen until a response was made. The participants were asked to maintain their gaze directed to a fixation cross that was presented at the center of the screen for the entire duration of the trial (both cues and probe presentation) and during the intertrial interval of 1,500 ms. Eye movements were monitored through EOG recordings. The participants were told that cue stimuli were not relevant for the task performance, so they should be ignored. They were asked to quickly and accurately respond to the probe, indicating its location by pressing the key “5” or “8” on the lateral numeric pad of the computer keyboard with their right index and medium fingers when the probe appeared on the right or left side, respectively. To familiarize with the procedure and to train the participants to fixate on the central cross, they were presented with 16 practice trials.

During the execution of the experimental task, EEG was recorded. The TMS-EEG procedure consisted of three experimental blocks with 192 trials each: in one block, only EEG was recorded during the execution of the dot-probe task (ERPs, no-TMS block); in two blocks, TMS pulses were applied 140 ms after cue presentation, and EEG was simultaneously recorded (TEPs, TMS blocks). The order of blocks was counterbalanced across participants.

### TMS Stimulation

TMS was delivered with an eXimia TMS stimulator (Nexstim, Helsinki, Finland) using a focal bipulse, figure-eight 70-mm coil. TMS targets were identified in each participant on a high-resolution 3D volume (3D magnetization-prepared rapid gradient-echo) acquired on a 1.5 T magnetic resonance scanner by means of a navigated brain stimulation (NBS) system (Nexstim, Helsinki, Finland) that uses a 3D infrared-based frameless stereotactic technology to map the position of the coil and the participant’s head within the reference space of the individual’s MRI space. TMS was applied 140 ms after cue presentation on the right FEF, localizing it above the junction of the precentral sulcus and superior frontal sulcus (mean coordinates: *X* = 32, SD: 4.3; *Y* = 18, SD: 10.6; *Z* = 57, SD: 4.7), based on the montreal neurological institute (MNI) template. The timing of the TMS pulse was defined on the basis of previous studies showing an early contribution of the frontal cortex in top-down modulation of the activity in posterior cortical regions (Morishima et al., 2009; Mattavelli et al., 2013). TMS was delivered at a mean intensity of 53.8% (range: 42%–62%) of the maximal stimulator output corresponding to an induced electric field of 97.3 V/m (range: 80–100 V/m). To prevent auditory potentials due to the TMS pulse, a masking noise, which reproduced the TMS “click” in time-varying frequency components, was continuously played into earplugs worn by participants during the experimental sessions (Massimini et al., 2005; Rosanova et al., 2009).

### EEG Recording and Analyses

EEG was continuously recorded using a TMS compatible 64-channel amplifier (Nexstim Ltd., Helsinki, Finland), which gated the TMS artifact and prevented saturation by means of a proprietary sample-and-hold circuit (Virtanen et al., 1999). The reference and ground electrodes were placed over the forehead, and electrooculograms were recorded with two additional electrodes placed near the eyes to monitor ocular artifacts. Electrode impedance was kept below 5 kΩ, and EEG signals were recorded with a sampling rate of 1450 Hz. EEG data preprocessing was carried out in MATLAB 2012a (MathWorks). Data were downsampled to 725 Hz, and the continuous signal was split into trials between −800 ms and +800 ms from the TMS pulse and the corresponding time window for ERP blocks. Trials with artifacts caused by muscle activity, eye movement or blink were removed by a semiautomatic procedure (Casali et al., 2010). The signal was bandpass filtered between 1 Hz and 45 Hz. Bad channels were interpolated using the spherical spline interpolation function of EEGLAB (Delorme and Makeig, 2004). TEPs were then averaged, referenced and baseline corrected between −450 ms and −230 ms before the TMS pulse, corresponding to −310 ms and −90 ms before the onset of the visual stimuli. Independent component analysis (ICA) was applied to remove residual muscular and magnetic artifacts (Korhonen et al., 2011; Johnson et al., 2012). Trials were then divided on the basis of experimental condition (competition LH and no competition HH) and side of face presentation in the competition condition (left and right visual field). Thus, TEPs and ERPs were computed by averaging selected artifact-free single epochs for each condition. After artifact rejection, the mean number of accepted trials in each condition was as follows. In the ERP blocks, there were 85 (SD = 6.99) trials in the competition condition (FH), composed of 42 (SD = 3.58) faces presented in the left visual field and 43 (SD = 3.87) faces in the right visual field, and 85 (SD = 7.73) trials in the no competition condition (HH). In the TEP blocks, there were 161 (SD = 15.18) trials in the competition condition (FH), composed of 81 (SD = 8.12) faces presented in the left visual field and 81 (SD = 7.56) faces in the right visual field, and 161 (SD = 15.18) trials in the no competition condition (HH).

Cortical responses in different conditions were compared through a cluster-based permutation test (Maris and Oostenveld, 2007) implemented in the FieldTrip MATLAB toolbox for M/EEG analysis (freely available at http://fieldtrip.fcdonders.nl/; Oostenveld et al., 2011). ERP and TEP blocks were separately analyzed, and for each block, two comparisons were performed with whole head, cluster-based, dependent sample *t*-tests to test: (1) the effects of competition of social emotional stimuli over control stimuli (FH vs. HH); and (2) the effect of side of face presentation (left face vs. right face). This procedure solved the multiple comparisons problem by permuting the data and clustering them on the basis of temporal and spatial proximity. In particular, *n* data permutations are performed by shuffling the trial labels, and *t*-tests are then computed at each time point for each permutation. Samples with statistics corresponding to a *P*-value smaller than a critical value are clustered together on the basis of temporal and spatial adjacency. Cluster-level statistics are calculated by taking the sum of the *t*-values within every cluster. Finally, the cluster-corrected threshold is computed as the permutation distribution of the maximum cluster-level statistics (Maris and Oostenveld, 2007). In our analyses, for each comparison, 10,000 permutations were performed with a permutation-significance level of *p* = 0.05 for the time window between 0 and 500 ms from cue onset, corresponding to −140 and 360 ms from TMS pulse in the TEP blocks. The time window was selected to cover the whole duration of cue presentation.

## Results

### Behavioral Results

The participants’ mean error rate in the dot-probe task was 3% (SD = 1.0). Analysis of variance (ANOVA) performed on the percentage of errors, with *Target visual field* (left, right), *Congruency* (congruent, incongruent) and *TMS block* (TMS, no-TMS) as within-subject factors revealed no significant effects.

ANOVA on response times on trials with correct answers revealed a significant main effect of *Target visual field*, *F*_(1,14)_ = 5.57, *p* = 0.033 because the participants were faster to respond when the target appeared on the right side of the fixation point, independent of the type and location of the preceding cue [right target mean reaction time (RT) = 492.5 ms, SD = 24.6; left target mean RT = 507.8 ms, SD = 28.9]. Other main effects and interactions were not significant (all *p*s > 0.05; Table 1).

**Table 1:** Mean reaction times (RTs; ms) and standard deviations from the dot-probe task in TMS (TMS evoked potentials, TEPs) and no-TMS (event related potentials, ERPs) blocks, in congruent, incongruent and control trials with target appearing on the left and right side of the visual field.

	TMS block		No TMS block	
	Left target	Right target	Left target	Right target
Congruent	517.3 (112.1)	496.5 (83.8)	502.4 (112.0)	495.6 (114.3)
Incongruent	516.7 (113.2)	493.9 (84.5)	498.6 (130.2)	484.3 (109.9)
Control	518.0 (104.3)	503.4 (87.8)	510.6 (130.3)	501.6 (118.0)

### ERP Results

**Figure 2:**
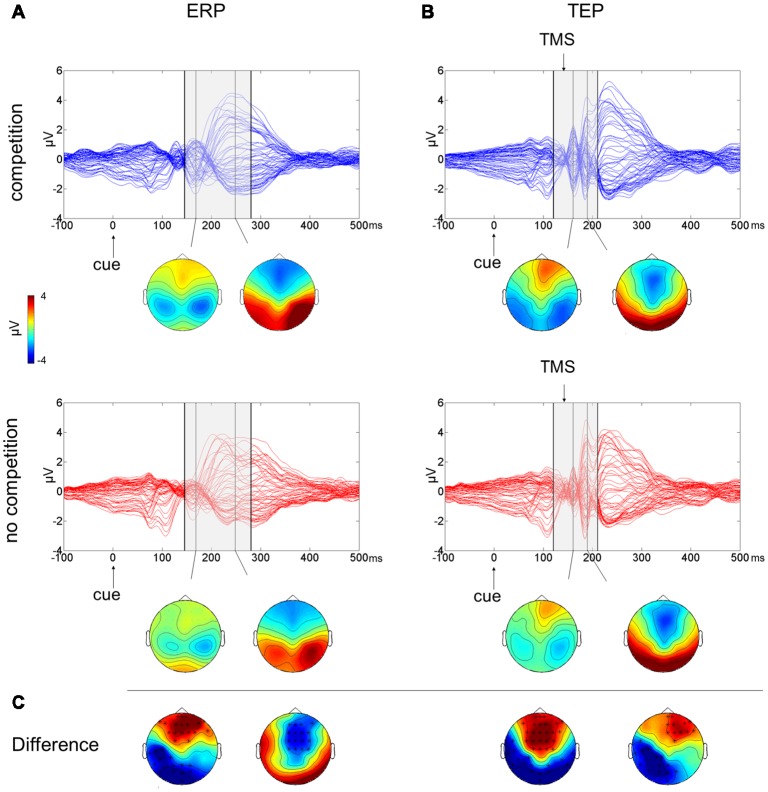
Multichannel butterfly plot and topoplot of the competition (blue trace) and no competition (red trace) conditions for average event related potentials (ERPs; **A**) and TMS evoked potentials (TEPs; **B**). The gray bars define the time window where cluster-based analyses evidenced significant results. Topoplots show the signal topography at waves’ peaks (ERPs: 170 ms and 240 ms; TEPs: 160 ms and 185 ms). Significant clusters are evidenced in the topoplot of the cluster analysis **(C)**.

The butterfly plots of the 60 channels superimposed (Figure 2A) show average ERPs recorded during the cue presentation of competing and not-competing pairs of stimuli with signals peaking at ~170 and ~240 ms.

A cluster-based analysis testing the effect of competition of social emotional stimuli (FH) over control stimuli (HH) revealed a significant positive cluster (*p* = 0.038; FH > HH) in anterior electrodes from 160 ms to 210 ms after cue onset and a significant negative cluster (*p* < 0.001; FH < HH) from 145 ms to 280 ms after cue onset. Scalp topographies of statistically significant differences (Figure 2C) showed that the positive cluster included fronto-central electrodes, shifting to right frontal electrodes at ~190 ms, whereas the negative cluster started in posterior left lateralized temporoparietal and occipital electrodes, moving at ~240 ms to fronto-central electrodes.

**Figure 3:**
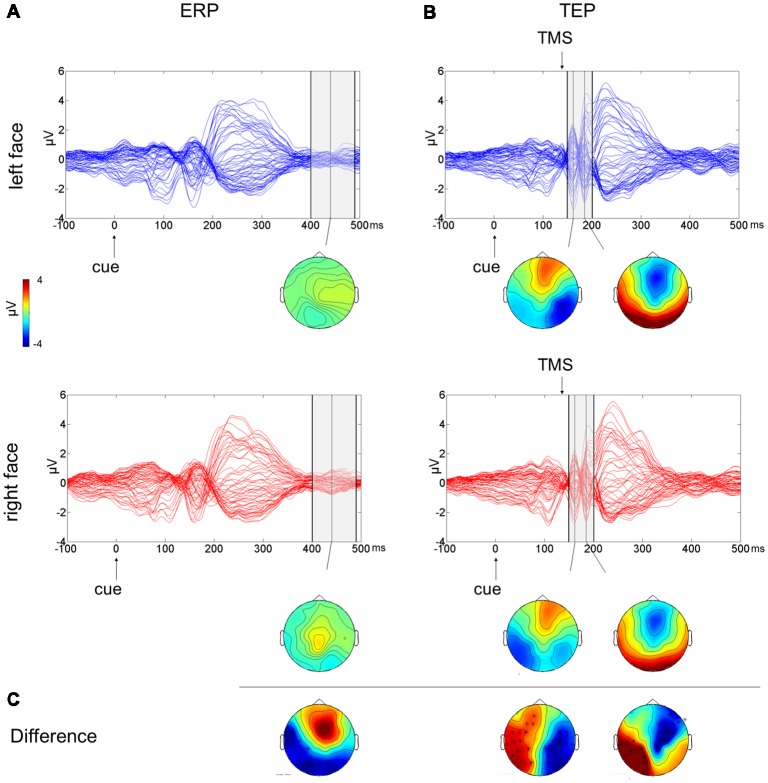
Multichannel plot and topoplot of the left face (blue trace) and right face (red trace) conditions for ERPs **(A)** and TEPs **(B)**. The gray bars define the time window where cluster-based analyses evidenced significant results. Topoplots show the signal topography at waves’ peaks (ERPs: 440 ms; TEPs: 160 ms and 185 ms). Significant clusters are evidenced in the topoplot of the cluster analysis **(C)**.

To test the effect of the side of presentation of the facial stimuli [face on left side (L) vs. face on right side (R)], a cluster-based analysis was performed considering only the competing condition of face-house stimuli (Figures 3A,C). This revealed a significant negative cluster (*p* = 0.018; L < R) from 400 ms to 490 ms from cue onset with posterior left lateralized topographical distribution.

### TEP Results

The butterfly plot in Figure 2B shows average TEPs recorded during the cue presentation of competing and not-competing pairs of stimuli. Two peaks at ~160 and ~240 ms from cue onset are shown (corresponding to ~20 and ~100 ms from TMS pulse) with latencies similar to ERP results but with amplified and better-defined EEG signals, and another peak is visible at ~185 ms from cue onset (~45 ms from TMS). A cluster-based analysis testing the effect of competition of social emotional stimuli (FH vs. HH) revealed a significant positive cluster (*p* < 0.001; FH > HH) in anterior electrodes from 120 ms to 195 ms from cue onset and a significant negative cluster (*p* = 0.006; FH < HH) in posterior electrodes from 130 ms to 210 ms from cue onset. TEP scalp topographies of statistically significant differences (Figure 2C) showed that the positive cluster was associated with fronto-central electrodes, whereas the negative cluster moved from bilateral temporoparietal and occipital electrodes to left lateralized temporoparietal electrodes.

The cluster-based analysis testing the effect of the side of presentation of the facial stimuli in the competition condition (L vs. R) revealed a positive cluster (*p* = 0.013; L > R) and a negative cluster (*p* = 0.004; L < R), both lasting from 150 ms to 200 ms from cue onset (from 10 ms to 60 ms from TMS pulse). The average butterfly plot and the scalp topographies of significant differences show the presence of two peaks in this time window at ~160 ms and at ~185 ms from cue onset (Figures 3B,C). Faces presented in the left visual field produced a greater negativity at right lateralized temporoparietal electrodes and greater positivity at left lateralized temporoparietal electrodes.

### Association Between ERPs/TEPs and Behavioral Results

A correlational analysis was run between the behavioral results and neurophysiological measures in the time windows where cluster-based analyses evidenced significant results in ERPs/TEPs blocks. In particular, as an index of the behavioral performance, we computed a bias score for each participant, calculated by subtracting RTs of congruent trials from RTs of incongruent trials (bias > 0 indicating faster responses in congruent condition). As neurophysiological measures, we computed an index of global cortical excitability, namely, the global mean field power (GMFP; for the formula used, see Lehmann and Skrandies, 1980; Romero Lauro et al., 2014), on ERP and TEP signals. The GMFP was computed on 60 channels in the time windows that significantly differed between conditions according to the cluster-based analyses. The GMFP of the no competition condition was then subtracted from the GMFP of competition condition (ΔGMFP).

For ERPs, the ΔGMFP was calculated in a time window from 160 ms to 210 ms for the positive cluster and from 145 ms to 280 ms for the negative cluster. The correlations between the bias score and the ΔGMFP were not significant in either the positive (*p* = 0.98) or negative (*p* = 0.85) clusters.

**Figure 4:**
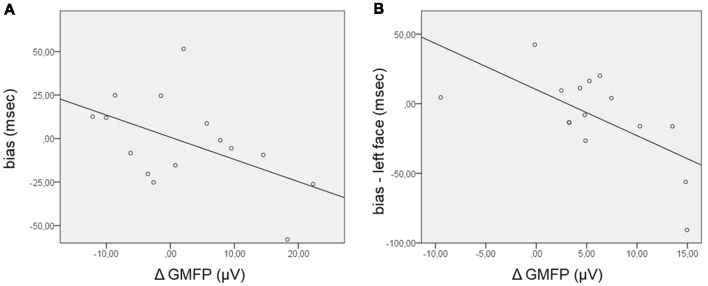
Scatterplot of the correlations between the bias score and the Δglobal mean field power (ΔGMFP) in the significant time window of the TEP cluster analysis. **(A)** ΔGMFP refers to the difference in GMFP in the competition and no competition conditions in the time window of the positive cluster (120–195 ms from cue onset); bias (ms) refers to the difference in RTs in incongruent − congruent trials. **(B)** ΔGMFP refers to the difference in GMFP for left and right face presentation in the time window of the positive and negative clusters (150–200 ms from cue onset); bias refers to the difference in corrected RTs in incongruent − congruent trials for left face cues.

Concerning the TEPs, the ΔGMFP was calculated in the time window from 120 ms to 195 ms for the positive cluster and from 130 ms to 210 ms for the negative cluster. The relationship between the bias score and the ΔGMFP showed no significant correlation in the negative cluster time-window, whereas a trend to significance (Pearson’s *r* = −0.51; *p* = 0.054) emerged, correlating the bias score with the ΔGMFP in the time window of the positive TEP cluster (Figure 4). The negative correlation indicates that an increasing global cortical excitability following right FEF TMS pulses in the competition condition (FH) was associated with more negative bias scores, i.e., with faster RTs for incongruent than congruent trials.

Additional analyses were performed focusing on the side of presentation of the face during the competing condition. RTs were first corrected for the effect of facilitation found when the target was presented in the right visual field by subtracting RTs in the control condition (HH) from RTs in the competition condition (FH), separately for congruent and incongruent trials and for the side of face presentation [for example: corrected left face congruent = left face congruent (left target) − left target control; corrected left face incongruent = left face incongruent (right target) − right target control]. The corrected bias for the left and right face cues was then calculated by subtracting corrected congruent RTs from incongruent RTs separately for the left and right face presentations. In this case, the GMFP was computed for ERP and TEP clusters separately for the condition of left and right face presentation and then subtracted (left − right) within the time windows where cluster-based analyses comparing the side of face presentation evidenced significant results. Regarding ERPs, no significant correlation between the ΔGMFP in the time window from 400 ms to 490 ms and the corrected bias for either the left or right face was found. Confirming the trend for the relationship between the performance in the behavioral task and the neurophysiological measure in the TMS blocks, the corrected bias score significantly correlated with the ΔGMFP of TEPs in the time window from 150 ms to 200 ms (Pearson’s *r* = −0.64; *p* = 0.011) when faces were presented in the left visual field (Figure 4), whereas in the case of right face presentation, correlations were not significant (Pearson’s *r* = −0.106, *p* = 0.706). This finding indicated that when faces were presented in the left visual field, an increase in cortical excitability following right FEF TMS was associated with more negative bias scores, i.e., faster RTs for incongruent than congruent trials.

## Discussion

A key unresolved challenge for neural and cognitive models of social processing is to clarify how the processing of social signals prevails over other stimuli. In the natural environment, social stimuli are very frequent, highly salient, and compete with nonsocial stimuli to gain access to perceptual awareness. It is therefore adaptive that they receive priority in information processing. Indeed, the existence of attentional biases for social stimuli such as happy and angry faces is well documented both in the general and clinical population (Langton et al., 2008; Browning et al., 2010a), suggesting an interplay between social perceptual processing and attentional orienting (Carretié et al., 2004; Frischen et al., 2007).

In this study, we examined modulation of cortical excitability by means of combined TMS-EEG during the early phases of unintentional emotional attention orienting, triggered by the competition between task-irrelevant lateralized stimuli. The right FEF was stimulated with TMS during the cueing phase of a dot-probe task to investigate its role in orienting attention towards socially relevant stimuli such as emotional faces. The main objective of the present study was to explore FEF excitability and the distribution of the neurophysiological response during the presentation of competing stimuli. Then, we investigated whether changes in the excitability were associated with the behavioral performance.

The dot-probe paradigm did not reveal any significant attentional bias; nevertheless, we found that the enhanced cortical excitability induced by TMS over the right FEF significantly correlated with the performance at the behavioral task, suggesting a link between the FEF activity during the cueing phase of the dot-probe task and the subsequent response times to targets. Our ERP findings revealed more pronounced anterior positivity and posterior negativity peaking at ~170 ms from cue onset for competing social stimuli compared to neutral pair of stimuli and an enhanced anterior negativity until ~270 ms from cue onset. In addition, the effect of the presentation side of face stimuli resulted in a late posterior negativity at ~400–500 ms from cue onset ipsilateral to the face presentation. TMS applied to the right FEF unveiled amplified early EEG components for competing cues similar to ERPs, which appeared related to the amount of attentional bias observed in the behavioral task. Moreover, when comparing the presentation side of face stimuli, TEPs showed a lateralized negativity at ~150–200 ms from cue onset contralateral to faces presented in the left visual field, which significantly correlated to the behavioral bias.

The early EEG components modulated in the comparison between competing and control pairs of stimuli, both in the ERP and TEP results, were consistent in terms of time range and topographical distribution with the N170 posterior negativity and the vertex positive potential (VPP), its analogous in latency peaking over fronto-central areas for face processing. N170 is known to be related to structural encoding and holistic processing of faces (Rossion and Gauthier, 2002). A large body of literature exploring the influence of emotional expression on this ERP component has reported conflicting results (Eimer and Holmes, 2007; Leppänen et al., 2007); however, the recent meta-analysis by Hinojosa et al. (2015) supports the notion that emotional faces elicit larger N170 than neutral faces. In line with previous literature, we found larger negativity in the competition condition, namely, when a face was presented compared with the control condition with two house stimuli presented. The effect of emotional valence on electrophysiological components was beyond the aims of the present study since the expression of anger was used only to reinforce the social meaning of facial stimuli in competition with neutral stimuli such as houses. Nevertheless, the choice of emotional faces could have contributed to the observed effects in the time window approximately 150–200 ms from cue onset.

A similar pattern of results emerged following the perturbation of the right FEF during the early cueing phase of the dot-probe task. Indeed, enhanced posterior negativity and anterior positivity in the time range of 140–200 ms were observed for competing cues also in TMS blocks. Interestingly, there was a trend towards a correlation between the cortical excitability measured following FEF perturbation and a disadvantage in responding (higher RTs for congruent than incongruent trials) to targets appearing at the same location of angry faces. This finding revealed that the excitability of the right FEF at 140 ms from cue onset was modulated by the presence of social stimuli, thus corroborating its involvement in attentional processing and suggesting a relation between the electrophysiological and behavioral data.

The early involvement of the prefrontal cortex in top-down attentional processes has been reported in previous studies. Frontal cortical signals precede attentional enhancement in the parietal and visual cortex and contribute to top-down processing in a time window of approximately 100 ms (Mattavelli et al., 2013; Kehrer et al., 2015; Banerjee et al., 2017). A TMS study investigating response priming in spatial conflict indicated that the right FEF plays a critical role in a time window between 80 and 120 ms after the onset of a visual stimulus (Bardi et al., 2012). Morishima et al. (2009) applied TMS over right FEF at 134 ms from target onset in a visual selective attention task and found that the change in TMS-induced activation in different posterior visual areas was dependent on the domain of visual features to which participants attended. In particular, the authors described how FEF stimulation induced an increase in activation in the fusiform face area (FFA) in a face-processing condition, whereas it induced an increase in the activity in the visual motion sensitive area in a motion-processing condition.

The ERP results showed that a negative cluster in anterior electrodes relative to the competing pair of stimuli was extended until 280 ms from cue onset. This finding appears consistent in terms of time range and topographical distribution with the N200 component, which has been described as generated from regions of the medial frontal cortex and associated with cognitive and attentional control processes (Dennis and Chen, 2007; Folstein and Van Petten, 2008). It has been reported that N200 is enhanced during conflict processing (Van Veen and Carter, 2002; Nieuwenhuis et al., 2003) and may reflect both the effort to inhibit attention towards fearful faces and the reduced resource availability for attentional tasks (Dennis and Chen, 2007). Thus, the enhanced N200 component observed in the present study during the presentation of competing “angry face-house” pairs may be the result of the need for increased cognitive control necessary for processing conflicting information. This is also in accordance with the notion of a “general alerting system” expressed by frontally generated ERP components (Suwazono et al., 2000) as a gating mechanism in favor of motivationally significant information (Van Veen and Carter, 2002; Nieuwenhuis et al., 2003).

While the effect of competition similarly modulated ERP and TEP results, a different pattern of results emerged when considering the effect of lateralization of the social and emotional stimuli in the competition condition. Specifically, ERPs revealed a posterior negativity ipsilateral to the side of face presentation in a late time window after 400 ms from cue onset. On the other hand, TEPs revealed a lateralized negativity for contralateral face stimuli and a lateralized positivity for ipsilateral face stimuli, both observed at 150–200 ms from cue onset.

Interestingly, the topographical distribution and waveforms observed in the ERP results have some commonalities with the EDAN component described following symbolic cues directing attention to left and right lateral locations. EDAN and ADAN components have been proposed to reflect the orienting of attention and then the holding of attention to the location indicated by a cue (Nobre et al., 2000; Eimer et al., 2002). Previous studies have explored whether social cues such as gaze direction could elicit EDAN and ADAN components similarly to arrow cues and have reported contrasting results (Hietanen et al., 2008; Holmes et al., 2010; Lassalle and Itier, 2013). Differences with respect to the typical features of the EDAN in terms of timing and lateralization could be ascribed to a variety of factors since in this study, we presented two lateralized cues instead of a single central cue, representing faces and houses instead of manipulating the direction of arrows or gaze, with faces expressing emotional valence. However, the negativity over the posterior electrodes at 400–490 ms from cue onset related to the side of presentation of face stimuli found in the present study may indicate EEG correlates similar to EDAN component reflecting orienting mechanisms of visuospatial attention.

Crucially, when cortical excitability was probed with TMS applied over FEF, the effect of face side presentation was revealed within 60 ms from the TMS pulse, corresponding to 150–200 ms from cue onset. Specifically, we found that the TMS on the right FEF induced an enhanced negative deflection in the right hemisphere when angry faces were displayed contralaterally and a reduced negative deflection in the left hemisphere when faces were displayed ipsilaterally. This early component from the TMS pulse reflects cortical excitability of the stimulated area (Pisoni et al., 2018), and it is consistent with the N1 component, which is a sensory component evoked by a visual stimulus and also reflects the engagement of sensory gating mechanisms of attention (Wascher et al., 2009; Chen, 2012). Previous research has also demonstrated a modulation of this component in the processing of socially relevant stimuli, showing greater N1 amplitudes for emotional than neutral stimuli (Foti et al., 2009; Carretié, 2014). Moreover, we found that the increase in cortical excitability following TMS pulses on the right FEF for faces presented in the left hemifield was significantly associated with the behavioral performance for left congruent trials (targets at the same location of face cue). These findings suggest that the right FEF plays a key role in the modulation of early EEG components, most likely reflecting the processing of contralateral socially and emotionally relevant stimuli. This is also consistent with previous research showing that the FEF has a causal influence over the modulation of visual activity in posterior areas when attention is being allocated (Taylor and Thut, 2012). Short trains of TMS applied over the right FEF during the cueing phase of a visuospatial orienting task lead to greater negativity in the ipsilateral posterior electrodes until 200 ms after visual stimulus presentation (Taylor et al., 2006).

The present findings could be further supported by time frequency analyses investigating cortical connectivity to verify whether the spectral components reflect the topographical changes observed here. Accordingly, other TMS-EEG studies have focused on right FEF involvement in attentional control via modulation of alpha oscillations (Capotosto et al., 2009; Sauseng et al., 2011). Marshall et al. (2015) found that left and right FEF are causally involved in the attentional top-down control since inhibitory theta burst stimulation to the FEF resulted in a reduction of alpha modulation in the hemisphere contralateral to stimulation and in enhanced gamma modulation in the left visual cortex following right FEF TMS.

Pourtois and Vuilleumier (2006) reported on EEG and functional imaging studies using a dot-probe paradigm with emotional face cues. Their focus of analysis was both on the target and on the cueing phase. Although the main aim of the authors concerned the brain responses time-locked to targets induced by preceding emotional faces, they also analyzed brain responses related to the pairs of faces. ERP analyses revealed that fearful faces enhanced early visual components peaking at 90 ms from cue onset, whereas fMRI analyses showed that fearful but not happy faces increased the neural response of the medial occipitoparietal cortex, contralateral to stimuli presentation, suggesting an alerting effect triggered by fearful faces (Pourtois et al., 2004, 2006).

The FEF is a part of a distributed fronto-temporo-occipital neural circuit engaged in face perception and emotion processing (Vuilleumier and Pourtois, 2007). Here, we show that this region plays a key role in the early processing of emotional stimuli when they compete with other neutral stimuli. According to the biased-competition theory of attention, objects in the visual field compete for neural representation, and top-down signals modulate the integrated activity of connected brain regions to bias attention (Desimone and Duncan, 1995; Beck and Kastner, 2009). In line with this theory, the arousal-biased competition model proposes that both bottom-up and top-down factors bias competition in favor of high priority stimuli, such as social and emotional stimuli (Mather and Sutherland, 2011), thus suggesting the existence of a large-scale brain network in which sensory, representational, motor and motivational inputs are combined to direct spatial attention. In this context, the brain regions involved in interference inhibition and emotional processing (such as the ACC, left inferior frontal cortex and amygdala) may interact with the frontoparietal attention network either to enhance the priority of processing emotional stimuli or reducing their distraction (Luo et al., 2014). Thus, the FEF and the posterior parietal cortex would combine spatial and salience information and modulate the activity of the posterior regions for faster perceptual processing and target detection (Mohanty and Sussman, 2013; Baldauf and Desimone, 2014).

The correlation that we found between the enhanced cortical excitability induced by TMS over the right FEF for competing stimuli and the behavioral performance further suggests a link between the FEF activity during the cueing phase of the dot-probe task and the subsequent response times to targets. Indeed, the increase in cortical excitability following right FEF TMS pulses was associated with faster RTs for targets replacing houses as opposed to angry faces, particularly when faces were presented on the left, contralaterally to the stimulation side. The enhanced excitability observed for face-house pairs during the cueing phase of the task thus reveals biased attentional processing, shown by faster responses for targets opposite to threatening cues.

These findings could suggest the occurrence of an avoidant behavior associated with an early enhanced FEF excitability induced by threatening contralateral stimuli. Accordingly, the vigilance-avoidance hypothesis proposes that threat-related attentional biases vary over time, with rapid initial vigilance for high threat cues, followed by avoidance of detailed processing in attempt to reduce anxiety (Mogg et al., 2004; Cooper and Langton, 2006; Torrence et al., 2017). Nevertheless, conclusions should be drawn with caution since our behavioral results did not reveal any significant attentional bias related to faces. This result could be ascribed to some methodological factors, such as the long stimulus onset asynchrony (SOA, 500 ms). A long SOA has been adopted to allow the study of early and late EEG components, but it could have influenced the behavioral results, possibly inducing the phenomenon of inhibition of return (Posner et al., 1985; Pan et al., 2017). A significant behavioral effect was the faster response to right lateralized targets, independent of the type and location of the preceding cue. This could be ascribed to a right lateralized bias induced by the position of the response keypad on the right side of the keyboard and the right-handedness of all participants (Rubichi et al., 2006), even though the response buttons were positioned in vertical line and the middle finger instead of the index finger was used for right targets to avoid the Simon effect (Simon and Wolf, 1963).

In the present experimental procedure, competing cue stimuli were composed of angry face-house pairs to produce a competition setting between socially relevant and neutral stimuli. The social relevance of faces was emphasized by the emotional valence of anger, which is known to trigger enhanced attentional orienting and faster involuntary responses (Hansen and Hansen, 1988; Vuilleumier, 2002; Hammerschmidt et al., 2017). Future studies could address whether the present findings are influenced by the emotional valence of faces studying different emotions; moreover, neutral face vs. house pair as competing cues could be used to verify whether social relevance of faces, not conveying any affective meaning, is sufficient to bias attentional orienting. Finally, potential confounding factors due to the less frequent exposure to face stimuli in comparison to houses and their repetition throughout the task cannot be excluded. In fact, behavioral results and neurophysiological responses could have been influenced by the occurrence of a novelty effect related to faces or by repetition priming effects. However, both competing and not competing conditions presented the same amount of stimuli repetition, so it is unlikely that eventual repetition suppression effects could have affected the observed differences in the EEG results. Further studies could definitely rule out the influence of these methodological factors, using face-face pairs as control stimuli and reducing the number of repetition of each stimulus.

In summary, using a TMS-EEG approach, we described the neurophysiological correlates of unintentional attentional processing when stimuli with high social value are opposed to neutral stimuli. Our findings suggest a role for the FEF in early attentional processing, in particular, affecting the N1 component related to lateralized threatening faces. These findings contribute to a better definition of the timing and neural correlates of social attention and lead the way to other TMS-EEG studies investigating the role played by different and interconnected brain regions. Furthermore, it could have important implications in the understanding of social cognitive processes in clinical populations such as schizophrenia and autism spectrum disorder, in which abnormal attentional processes towards social stimuli could result in impaired social functioning.

## Data Availability

The raw data supporting the conclusions of this manuscript will be made available by the authors, without undue reservation, to any qualified researcher.

## Author Contributions

ST designed the experiment, collected data, performed data analyses, interpreted the results and wrote the manuscript. ELG performed the experiment and edited the manuscript. GM designed the experiment, performed data analyses and revised the manuscript. LRL and RA-G contributed to the experimental design and revised the manuscript. PR conceived the study and revised the manuscript.

## Conflict of Interest Statement

The authors declare that the research was conducted in the absence of any commercial or financial relationships that could be construed as a potential conflict of interest.

## Footnotes

^1^http://agingmind.utdallas.edu/other-stimulus/
